# Signal amplification of a quartz crystal microbalance immunosensor by gold nanoparticles-polyethyleneimine for hepatitis B biomarker detection

**DOI:** 10.1038/s41598-023-48766-2

**Published:** 2023-12-09

**Authors:** Zahra Saffari, Reza Ahangari Cohan, Mina Sepahi, Mahdi Sadeqi, Mehdi Khoobi, Mojtaba Hamidi Fard, Amir Ghavidel, Fahimeh Bagheri Amiri, Mohammad Reza Aghasadeghi, Dariush Norouzian

**Affiliations:** 1 Nanobiotechnology Department, New Technologies Research Group, Pasteur Institute of Iran, Tehran, 1316943551 Iran; 2 Applied Biotechnology Research Center, Baqiyatallah University of Medical Sciences, Tehran, Iran; 3 Department of Radiopharmacy, Faculty of Pharmacy, Tehran University of Medical Sciences, Tehran, Iran; 4 Pharmaceutical Quality Assurance Research Center, The Institute of Pharmaceutical Sciences (TIPS), Tehran University of Medical Sciences, Tehran, Iran; 5 Hepatitis and AIDS Department, Pasteur Institute of Iran, Tehran, Iran; 6 Physics Department, Sharif University of Technology, Tehran, Iran; 7 Department of Epidemiology and Biostatistics, Research Centre for Emerging and Reemerging Infectious Diseases, Pasteur Institute of Iran, Tehran, Iran

**Keywords:** Antisense elements, Analytical biochemistry

## Abstract

The procedures currently used for hepatitis B (HB) detection are not suitable for screening, clinical diagnosis, and point-of-care testing (POCT). Therefore, we developed and tested a QCM-based immunosensor by surface modification with AuNP-PEIs to amplify the signal and provide an oriented-immobilization surface. The AuNP-PEIs were characterized by ICP-Mass, UV/Vis, DLS, FE-SEM, and ATR-FTIR. After coating AuNP-PEIs on the gold electrode surface, anti-HBsAg antibodies were immobilized using NHS/EDC chemistry based on response surface methodology (RSM) optimization. The efficiency of the immunosensor was assessed by human sera and data were compared to gold-standard ELISA using receiver-operating-characteristic (ROC) analysis. FE-SEM, AFM, EDS, and EDS mapping confirmed AuNP-PEIs are homogeneously distributed on the surface with a high density and purity. After antibody immobilization, the immunosensor exhibited good recognition of HBsAg with a calibration curve of ∆F =  − 6.910e^-7^x + 10(R^2^ = 0.9905), a LOD of 1.49 ng/mL, and a LOQ of 4.52 ng/mL. The immunosensor yielded reliable and accurate results with a specificity of 100% (95% CI 47.8–100.0) and sensitivity of 100% (95% CI 96.2–100.0). In conclusion, the fabricated immunosensor has the potential as an analytic tool with high sensitivity and specificity. However, further investigations are needed to convert it to a tiny lab-on-chip for HB diagnosis in clinical samples.

## Introduction

Hepatitis B virus (HBV), a prototypical member of the Hepadnaviridae family, is considered a noncytopathic hepatotropic virus, which can cause persistent infection (CHB, namely chronic hepatitis B). The virus is transmitted through perinatal or horizontal transmission (exposure to infected blood and body fluids). Liver cirrhosis and hepatocellular carcinoma (HCC) are the last pictures of the infection^1,2^. It should be noted that viral hepatitis, as a public health threat, has long been relevant all over the world. According to World Health Organization (WHO), About 260 million humans (> 3% world's population) suffer from CHB in endemic regions including Africa, Asia, and parts of Central and Eastern Europe. More importantly, approximately one million humans die annually from complications of CHB, liver cirrhosis, and HCC because of non-curative choices^1–3^. WHO recommends vaccination, early and accurate diagnostic detection, and education campaigns to break the chain of transmission and mitigate the disease burden impacts on communities? Since vaccination for HB is not applicable, timely diagnosis and education campaigns are crucial in preventing an increase in the number of people affected by the disease^1,4^.

Hepatitis B surface antigen (HBsAg) is a clinically important biomarker for the diagnosis and screening of acute or chronic states^5^. Enzyme-linked immunosorbent assay (ELISA) is the well-established gold standard for the detection of HBsAg in the blood.

However, this technique is a time-consuming procedure, needs large sample consumption, and requires two monoclonal antibodies. These shortcomings become more important by considering the delayed time between the patient referral and the diagnosis^5,6^. With recent advances in biomarker-based diagnostics, the development of affordable, sensitive, specific, user-friendly, rapid, and convenient methods for measuring analytes has become achievable. One of these promising and powerful platforms is biosensor technology^7–9^. In our previous studies, we developed the quartz crystal microbalance (QCM)-based immunosensor for HBsAg detection^6,10^ that benefits superior analytical advantages including sensitivity, selectivity, repeatability, fast response, and POCT^11–13^. Nonetheless, higher sensitivity is needed for early or chronic diagnosis of the disease. In this regard, signal amplification is considered an able-minded solution for designing and developing highly sensitive QCM biosensors^14–16^.

Recently, signal-amplification scaffolds have been employed for high-precision detection of small target analytes. Chemicals and biomaterials have been used as signal amplifiers in QCM biosensors such as nanoparticles, enzymes, and liposomes. Among them, nanoparticles show great potential in increasing sensitivity because of their large mass/volume ratio compared to most analytes and their capability for functionalization^17^. Inorganic nanoparticles like gold nanoparticles (AuNPs) are propounded as an appropriate option for this purpose^18–20^. AuNPs are popularly utilized for the development of QCM biosensors because of their unique properties including small size, a large surface area to volume ratio, high reactivity, stability over high temperatures, and tuning of the surface charge. Consequently, AuNPs have potential characteristics for signal amplification in biomarkers-based sensors. Moreover, AuNP synthesis is carried out in a simple and controllable manner^14,21,22^.

In this study, we devised a highly sensitive QCM-based immunosensor by layering AuNP-PEIs on the gold electrode for real-time detection of antigen–antibody interaction. Polyethyleneimine (PEI) was used as a reducing and stabilizing agent during AuNPs synthesis^19,23^. PEIs capped on AuNPs provide appropriate functionality for antibody immobilization, as well as intrinsic characteristics such as water solubility, pH buffering capability, and complex formation with metal ions^9^. The entire immunosensor construction process was characterized through frequency measurements and various physical and chemical methods including inductively coupled plasma mass spectrometry (ICP-MS), UV–Vis spectroscopy, dynamic light scattering (DLS), field emission scanning electron microscopy (FE-SEM), Fourier-transform infrared spectroscopy (ATR-FTIR), wetting contact angle (WCA), and energy-dispersive X-ray spectroscopy (EDS) mapping. We calculated the limit of detection (LOD) and limit of quantification (LOQ) of the developed QCM-based immunosensor and then tested it for the sensitive and selective detection of HBsAg in 100 human sera samples. Finally, we conducted ROC analysis and correlation studies to determine the optimal cut-off value for the developed immunosensor.

## Results and discussion

### AuNP-PEIs preparation

As mentioned, AuNPs have many advantageous properties, and importantly, have a useful role in amplifying signals and enhancing sensitivity when used as a component in biosensor design^21,24^. For example, AuNPs amplified the signals for monitoring of Pb^2+^ in drinking water^25^, *Escherichia coli* (*E. coli*)O157:H7 in foods^26^, CD10 marker in lymphoblastic leukemia^27^, leukemia cells in the blood^28^, and *Campylobacter jejuni* in foods ^17^ in QCM-based sensors. The conventional methods for the reduction of Au^3+^ ions include the use of sodium borohydride (Burst method)^29^ or sodium citrate (Turkevich method)^30^ as reducing agents. However, in the current study, we used PEI as the reducing, capping, and stabilizing agent to simultaneously synthesize and functionalize the AuNPs ^31,32^. The primary amines that existed on PEI stabilize AuNPs by capping and also act as functional groups for antibody immobilization. To the best of our knowledge, the preparation of AuNPs using PEI has not been previously reported as a linker in QCM-based immunosensors.

### Physicochemical characterization of AuNP-PEIs

**Figure 1:**
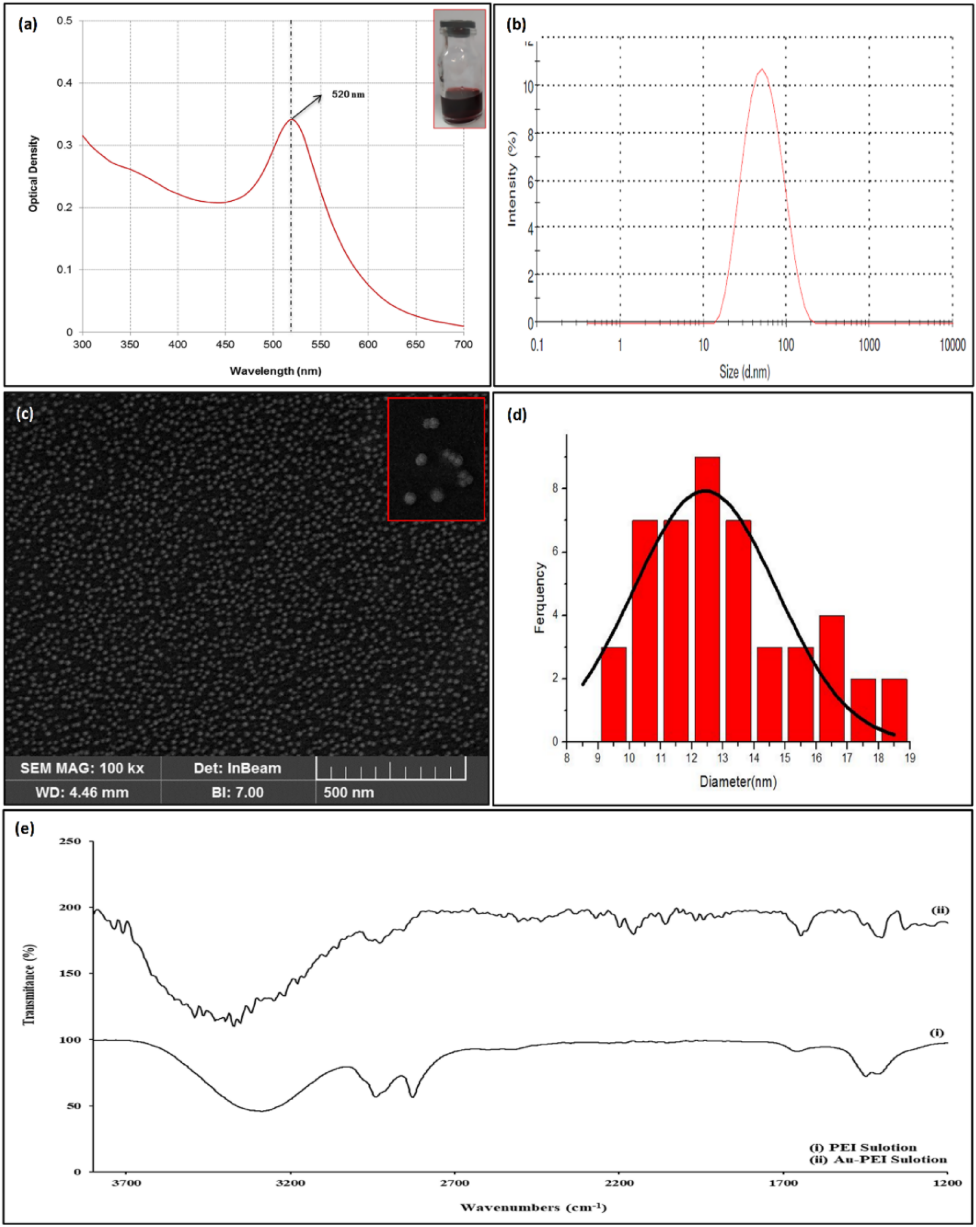
Characterization of the synthesized AuNP-PEIs (**a**) UV–vis absorption spectrum, (**b**) Particle size distribution with DLS, (**c**) Representative FE-SEM images, (inset: image with higher resolution), (**d**) Histogram for particle size distribution and Gaussian fitted curve, and (**e**) ATR-FTIR spectra of PEIs and AuNP-PEIs.

Physicochemical properties of the prepared AuNP-PEIs were characterized using ICP-MS, UV–vis spectroscopy, FE-SEM, DLS, and ATR-FTIR. At first, we used ICP-MS as the most versatile detection and elemental analysis technique that measures elements at milligrams to nanograms scale^33^. ICP-MS indicated the presence of gold in AuNP-PEIs dispersion. The concentration of gold in AuNP-PEIs was obtained at 323.78 µg/mL which was sufficient for further steps. UV–Visible spectroscopy was used to assure the synthesis and stability of AuNP-PEIs. A UV–Vis spectrophotometer equipped with an 8 + 8 cell changer recorded the spectra of the sample in a wavelength range of 300–700 nm. In the reduction process, Auº formation occurred after heating the PEI/Au^3+^ mixture. As seen in the UV–vis spectrum, an initial decrease in the absorption peak indicates the formation of Au^+^ at first, and over time, an increase in the absorption peak confirmed the formation of AuNPs^32^. UV–vis absorbance yielded a narrow surface plasmon resonance (SPR) band with a maximum wavelength (λmax) of 520 nm for the purple solution of AuNP-PEIs (Fig. 1a. inset), indicating a narrow size distribution and confirming the synthesis (Fig. 1a). Relevant studies have also shown similar λmax for AuNP-PEIs. In a study, various branched PEIs were used for AuNP synthesis with the same λmax (522 nm)^20^. However, in one report that used the Turkevich method for AuNP synthesis, a λmax of 524–534 nm was obtained after the conjugation of AuNPs with different branched PEIs^9^. This difference in the λmax can be explained by the different applied preparation methods and/or different sizes of branched PEIs used for AuNP-PEIs synthesis. So, the AuNP-PEIs formation was confirmed based on a SPR peak at ~ 525 nm^31^. There is a simple relationship between the particle size of AuNPs with UV–vis absorption spectra according to the Mie Theory^34^. In this regard, the ratio of the absorbance at the SPR peak (A_spr_) to the absorbance at 450 nm (A_450_) was calculated at 1.63. This estimates an average particle size of 15 nm for the prepared AuNP based on the UV–vis spectrum. This estimation was consistent with FE-SEM data.

The particle size distribution (d.nm) and zeta potential (mV) of the prepared AuNP-PEIs were measured in a batch mode by DLS. As shown in Fig. 1b, AuNP-PEIs had a monomodal hydrodynamic size distribution with a z-average of 43.16 nm and a mean polydispersity index (PDI) of 0.25. Based on the previous reports, a higher Mw for PEI more effectively maintains the colloidal stability of AuNP-PEIs^9^. The measured zeta potential of AuNP-PEIs was + 44.4 mV which corroborated the presence of amine-terminated surface coronas surrounding the gold core in the AuNP-PEIs. The positive charged AuNP-PEIs are convenient platforms for electrostatic coating on the gold electrode surface, and on the other hand, they provide a good capability for binding with the activated antibodies.

The surface morphology and size distribution of AuNP-PEIs were assessed using FE-SEM. FE-SEM images indicated AuNP-PEIs have a spherical shape (Fig. 1c. inset) and are extremely dispersed at the nanoscale (Fig. 1c). The histogram analysis of diameter revealed AuNP-PEIs have a narrow range size between 10 and 14 nm. Gaussian fitted curve indicated nanospheres with an average size of 12.43 nm (Fig. 1d). These observations were consistent with the narrow size distribution based on DLS data (a PDI value of 0.25) and the narrow SPR band based on UV–vis spectroscopy. It should be notified that the size distribution obtained by FE-SEM is smaller than that of DLS. It is worth mentioning that the DLS technique measures the hydrodynamic radius of the particles which is more important for biological functions^33^.

We examined the ATR-FTIR spectra of the AuNP-PEIs and PEIs (Fig. 1e). This spectroscopy illustrates the gold metal and polycationic polymer interaction. ATR-FTIR spectra of the samples displayed a similar pattern with negligible shifts. The broad band at 3000–3400 cm^-1^ could be devoted to the stretching vibration of the NH_2_ group of the primary amine of PEI. Meanwhile, the peaks between 2700 cm^-1^ and 3000 cm^-1^ could be attributed to the asymmetric and symmetric vibrations of aliphatic C-H bonds. The peaks related to the bending vibration of N–H and C-H bonds of PEI could be seen at 1660 cm^-1^ and 1450 cm^-1^, respectively, and showed a shift to nearly 1640 cm^-1^ and 1435 cm^-1^, respectively, after the addition and reduction of Au^+3^ ions^20^.

Stabilizing effect of PEI on the shelf-life of AuNP-PEIs was monitored within 4 months under optimum conditions by DLS and UV–vis spectroscopy. It has been reported that high-MW and branched PEI make the prepared nanoparticles more stable^19^. AuNP-PEIs showed a negligible shift in the z-average size (Δdz ≈ 7 nm) and a small change in the Δλ_max_ (5 nm redshift) after 4 months. It must be notified that after 4 months, the purple color of the solution was changed to red color, indicating an increase in the particle size due to the aggregation of the nanoparticles. These results were consistent with the previous studies^19,20^.

### Coating and characterization of AuNP-PEIs on QCM surface

Since the highest mass sensitivity occurs at the center of the electrode^35^, therefore, AuNP-PEIs were placed at the center of the cleaned chip and incubated at optimum conditions (24 h, RT). AuNP-PEIs electrostatically interacted via the primary amines with the cleaned gold electrode of the QCM**-**based immunosensor^36^. Then, FE-SEM, EDS, EDS Mapping, and AFM were used to study the topographical microstructure and elemental mapping of the modified gold electrode surface. In the following, the surface stability and hydrophilicity, as static requirements, were checked after modifications. A bare gold electrode was used as a reference in all tests.

### Morphological characterizations

#### FE-SEM

**Figure 2:**
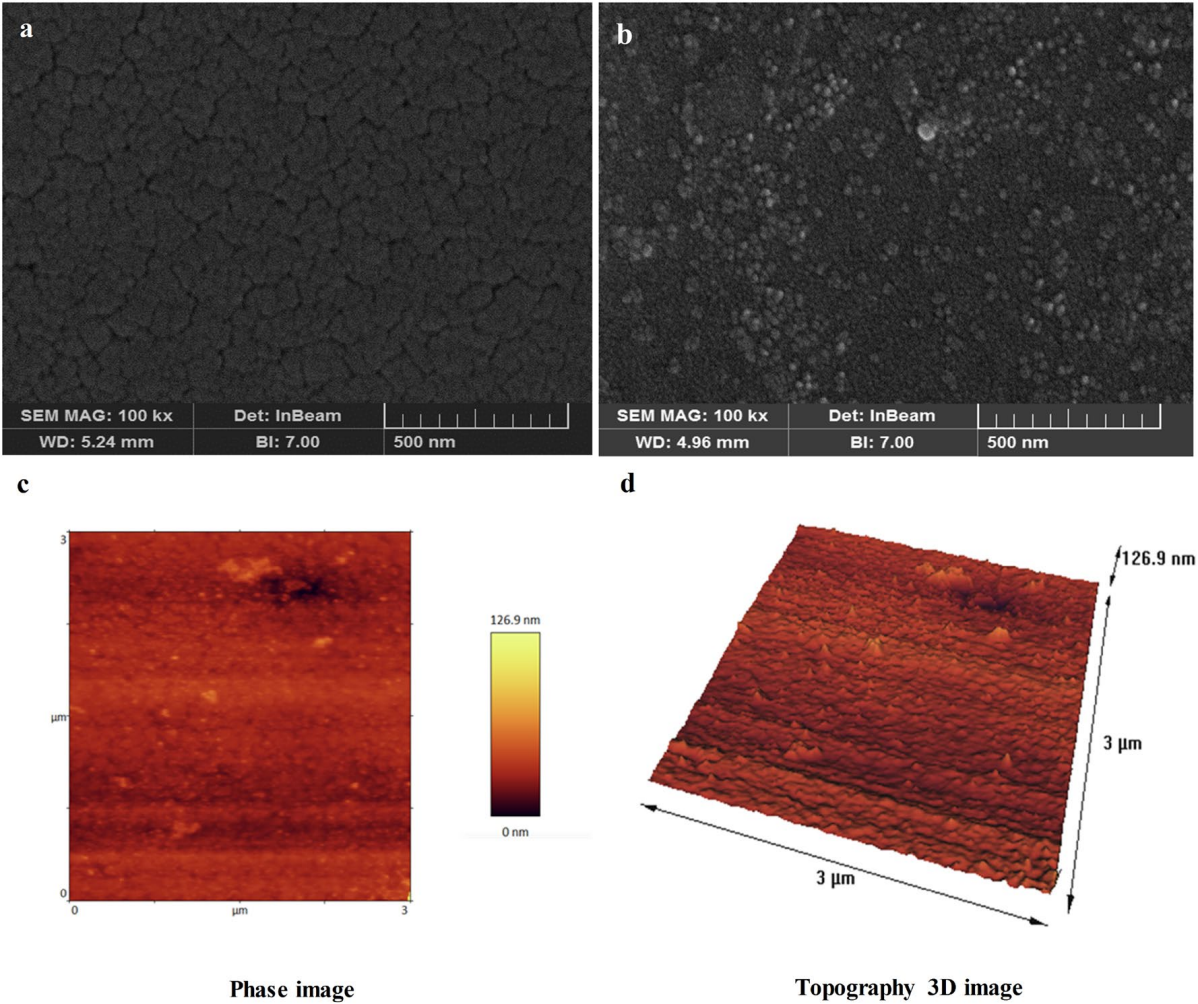
Morphological characterization of the modified electrode with AuNP-PEIs. (**a**) FE-SEM images of the reference electrode, (**b**) FE-SEM images of the modified electrode, (**c**) AFM phase imaging, and (**d**) 3D-topographic images acquired by AFM.

Figure 2 demonstrates the FE-SEM micrographs of the QCM surface before and after AuNP-PEIs coating. AuNP-PEIs coated surface (Fig. 2b) was characterized by a high level of granularity on the surface when compared to the reference (Fig. 2a). This characteristic provides a high surface/volume ratio for the conjugation of activated antibodies, where the covalent bonds occur between the NHS/EDC activated carboxyl groups of antibodies and the primary amines of AuNP-PEIs.

#### AFM study

The modified QCM surface was also evaluated by AFM as another microscopic tool. The AFM technique measures the change in the roughness of the modified surface and analyzes the uniformity of the layer over the entire area^37^. The thickness of the modified layer on the surface was determined using the non-contact mode of AFM by scanning a 3 × 3 μm square area at ambient pressure. Two-dimensional and three-dimensional topographic images of the modified surface were shown in Fig. 2c and d, respectively. As seen, AuNP-PEIs are uniformly dispersed on the gold electrode. The surface thickness after modification with AuNP-PEIs was 126.9 nm. The mass deposition was confirmed by an increase in the surface thickness compared to the reference electrode which was measured in our previous studies^6,10^. The average roughness was 2.38 ± 0.81 nm for the modified surface, while 1.15 ± 0.03 nm for the reference electrode, indicating an increase in the surface roughness due to the acceptable presence of PEI-capped AuNPs.

### Chemical characterizations

#### EDS, and EDS mapping

**Figure 3:**
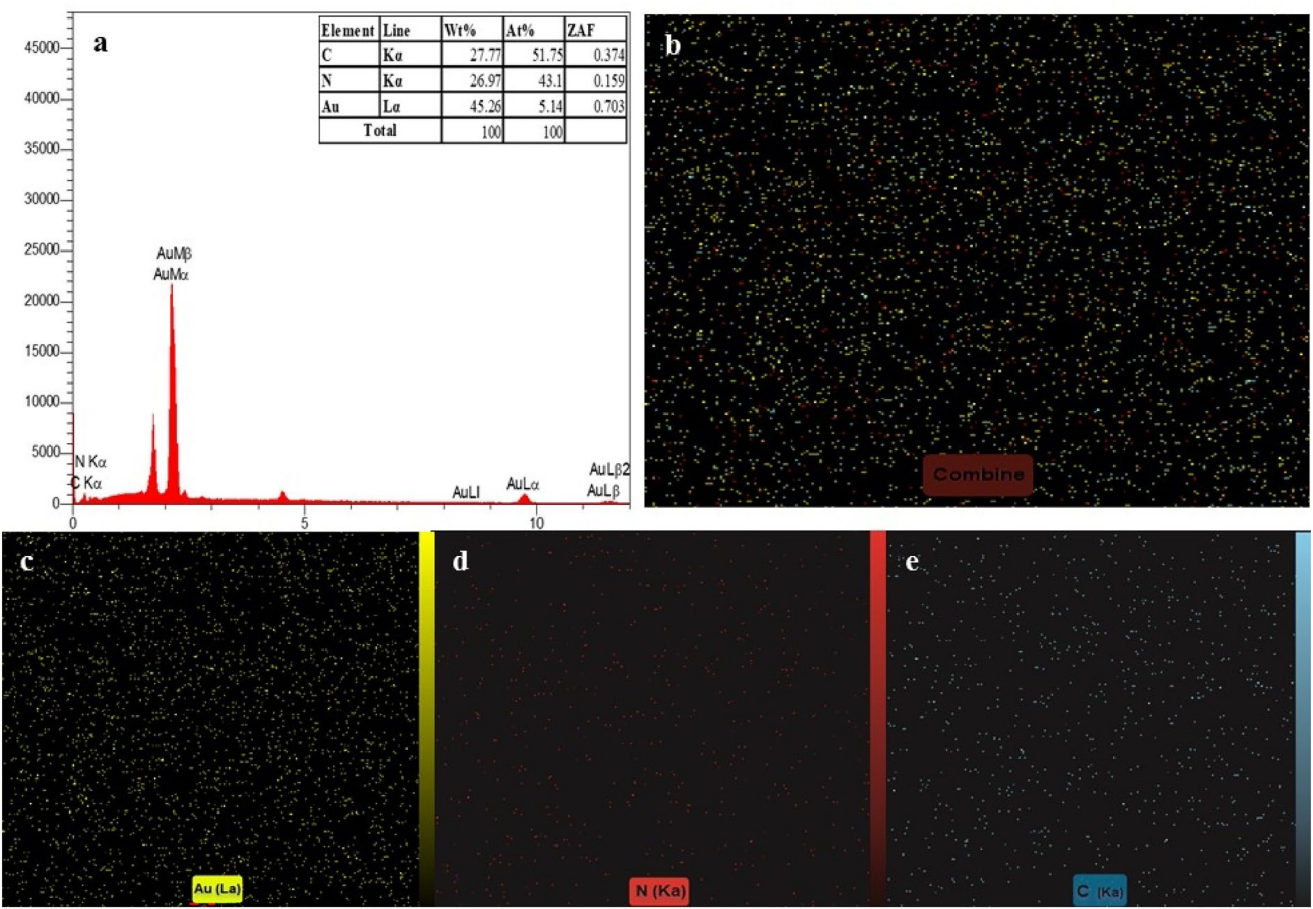
(**a**) EDS graph of the immobilized AuNP-PEIs on the surface of QCM, (**b**) EDS mapping of AuNP-PEIs, elemental EDS mapping of (**c**) gold (yellow color), (**d**) nitrogen (red color), and (**e**) carbon (blue color).

Also, the elemental composition of the modified QCM was examined by EDS, showing that the surface of QCM was successfully coated with AuNP-PEIs (Fig. 3a). EDS mapping showed that AuNP-PEIs were homogeneously distributed on the QCM surface with a high density similar to that seen in the FE-SEM images (Fig. 3b). The EDS mapping profile of gold (Fig. 3c), nitrogen (Fig. 3d), and carbon (Fig. 3e) also confirmed the immobilization of the NPs on the surface of QCM with high purity.

### Static characterization

#### Stability

**Figure 4:**
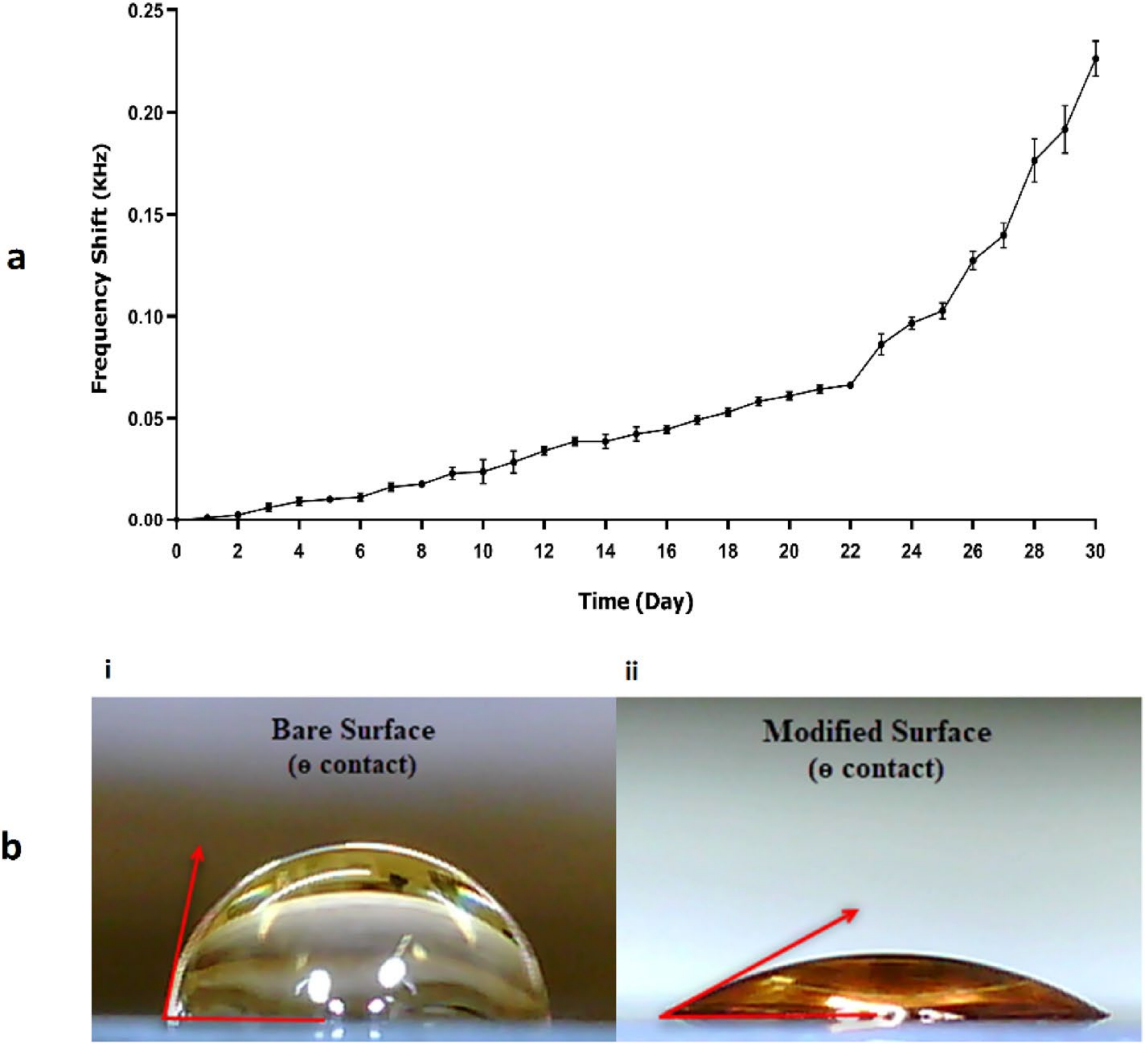
(**a**) The stability of the modified surface within 30 days. The data were represented as mean ± SD (n = 3). (**b**) The images of water drop on (**i**) the reference electrode and (**ii**) the modified electrode.

Investigation of static requirements is essential to optimize immunosensor for commercial use. One of the necessary characteristics for developing a highly efficient biosensor is the stability of infrastructures where the immobilization of receptors is needed. The environmental parameters can severely affect the layer's stability as well as the performance of antibody immobilization on sensors^12^. The highly humid atmosphere and ambient temperature changes directly affect the electrostatic interaction between the polymer layer and gold electrode surface via swelling, stretching, or peeling off the polymer from the surface, eventually damaging the sensor functionality. It is worth mentioning that precise control of temperature near RT is necessary for high-accuracy measurements with QCM. Therefore, controlling environmental conditions is essential for obtaining reliable and reproducible responses^38,39^. In this study, the useful lifetime of the modified layer was monitored for 30 days at RT. The AuNP-PEIs coating led to a frequency shift with a gradual increase till the 22nd day, and a sudden increase until the 30th day (Fig. 4a). Therefore, it can be concluded that in addition to the amplifier role, AuNP-PEIs have better stability than free PEIs. This significant increase in stability can be explained by the presence of more amine groups due to the large surface provided by AuNPs, which in turn creates stronger electrostatic interaction between the two surfaces.

#### Inner WCA

As revealed in Fig. 4b, the WCA (θ°) was measured between the sessile drops of WFI and the gold electrode surface before and after AuNP-PEIs layering using an optical camera (Dino-Lite, China) and digimizer (digimizer version 5.4.9, Medcalc Software Ltd). The WCAs were calculated at 76.13° ± 3.89 and 25.56° ± 1.96 for the reference and modified surface, respectively. According to the sessile drop technique results, the WCA decreased after the modification (~ 50°), which meant an increase in the hydrophilicity of the surface. These results are well-correlated with our previous studies^6,10^.

#### Monitoring the anti-HBsAg antibody immobilization on the QCM-based immunosensor

Antibody immobilization is a critical step in designing the recognition part for QCM-based immunosensor. The appropriate immobilization technique is necessary because there is a chance for the antibody to be leached away after immobilization. The popular method for coupling between the available functional groups on the antibody and the functional groups present on the surface is zero-length heterobifunctional EDC-NHS/sNHS chemistry, in which NHS/sNHS leaves the activated surface or molecule after an amide bond formation^40^.

The effect of antibody activation time in the EDC/NHS reaction (the time spent activating carboxyl groups on the antibody, referred to as Factor A) and the antibody immobilization time (the time spent forming an amide bond between the activated carboxylic groups and amine groups on the modified surface, referred to as Factor B) on the immobilization yield (referred to as a response) was modeled by a face-centered central composite design. The experimental matrix and the corresponding results of immobilization yields are presented in Table S_1_.

The software used analysis of variances (ANOVA) for predicting a quadratic polynomial equation to describe the relationship between independent variables (here A: antibody activation time and B: immobilization time) and the target response (here Y: Immobilization yield) as follows (Eq. 1):

1 $$ {\text{Y}} = - 88.723 + 10.185{\text{A}} + \, 0.445{\text{B}}{-}0.009\,{\text{AB}}{-}0.161{\text{A}}^{2} $$

The predicted model had a *p*-value of less than 0.05 and was significant (Table Table S_2_).

**Figure 5:**
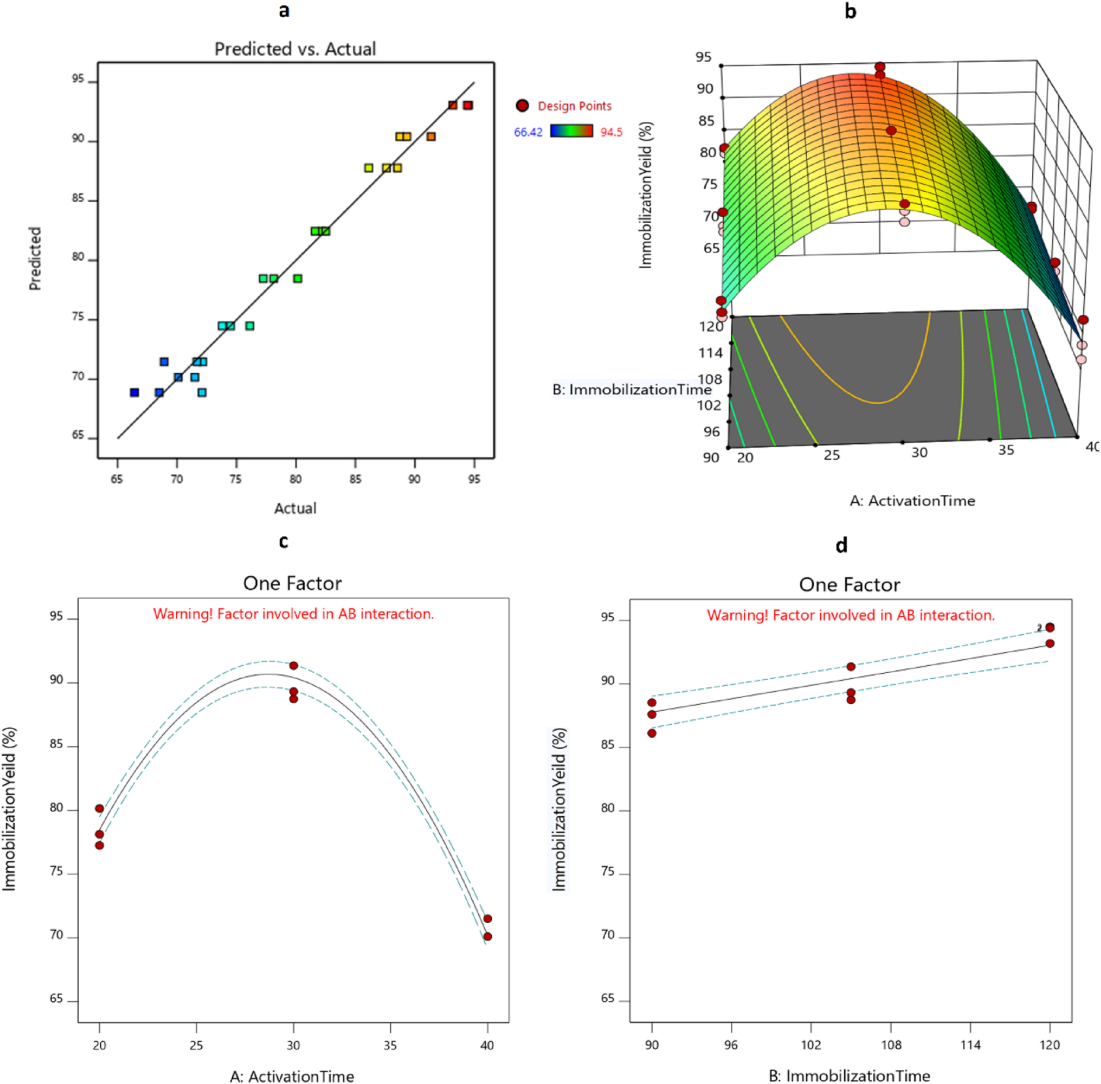
Modeling the effects of immobilization and activation times on the antibody immobilization yield. (**a**) Predicted versus actual plot, (**b**) the 3D-plot of the interaction of factors on immobilization yield, (**c**) the effect of activation time on immobilization yield, and (**d**) the effect of immobilization time on immobilization yield.

The determination coefficients (R^2^) and adjusted R^2^ were 0.9766 and 0.9722, respectively, indicating the adequacy of the model. The adequacy of the model was also evaluated by predicted versus actual plot (Fig. 5a). All points were scattered close enough to a diagonal line, indicating the predicted model fitted the empirical data adequately. Figure 5b shows the activation and immobilization times' interaction with immobilization yield. The effects of each factor on target response are shown in Fig. 5c and d.

With the aid of the predicted model, the activation and immobilization times were optimized using the optimization option of Design Expert software to achieve maximum immobilization yield. The optimum criteria were predicted as 30 min for antibody activation and 120 min for immobilization time, resulting in an immobilization yield of 93.5%. These predicted optimal criteria were similar to those of runs No. 2, 9, and 15. To investigate the accuracy of the predicted optimum criteria, the immobilization yields of mentioned runs were compared with the predicted values using a one-sample T-test with SPSS software (Table Table S_3_).

It has been reported that the heterobifunctional cross-link efficiency will affect the biosensor`s performance. This efficiency is well-correlated with the ratio and molar concentrations of EDC/NHS, as well as the time of activation, all of which vary from one study to another^41^. In the current study, we used the appropriate concentration/ratio of EDC/NHS as described in previous studies^42^, however, the yield of antibodies activation was investigated at different times. In a similar study conducted by Ehsani et al*.*, an anti-HBsAg antibody was conjugated either to CuO nanoparticles or CuO/MWCNT nanocomposites for the detection of HBsAg in biological samples. The effect of activation time on the formation of nano-conjugates was investigated within 1 h. The maximum intensity of detection (chemiluminescence immunoassay) was obtained at 40 and 30 min for CuO nanoparticles and CuO/MWCNT nanocomposites, respectively^43^. In our study, the optimal activation time was achieved below 1 h, even less than Ehsani et al*.* (20 min versus 30 and 40 min). This achievement may be explained by using RSM in our study. This strategy (antibody activation) is notable because the activation of bio-recognition elements occurs faster in solution than on surfaces. Usually, surface activation takes equal to or more than 1 hour^44,45^.

For example, a QCM-based sensor was developed to detect *E. coli* O157:H7 in samples. The cleaned QCM-based sensors were immersed in 16-mercaptopropanoic acid solution for 24 h, and the modified surfaces were treated with EDC/NHS for 2 h to convert the terminal carboxylic groups to active NHS esters. Despite the existence of carboxylic groups on the antibody and surface, the use of such a strategy (activation on the surface) was inescapable for them, because the amine groups were not present on the surface for the second step of coupling^44^. In addition, they utilized a constant time for immobilization of antibodies on the activated surface, which was 2 h. In another study, Asiaei et al*.* also utilized the EDC/NHS-activated self-assembled monolayer (SAM) (11-mercaptoundecanoic acid and 1-octane thiol) as a linkage layer for site-directed immobilization of antibodies to develop a Salmon HSP70 biosensor. At the optimal conditions, one-hour time was needed for the activation of the carboxyl groups by 5 mM EDC/NHS solution on the SAM^46^.

**Figure 6:**
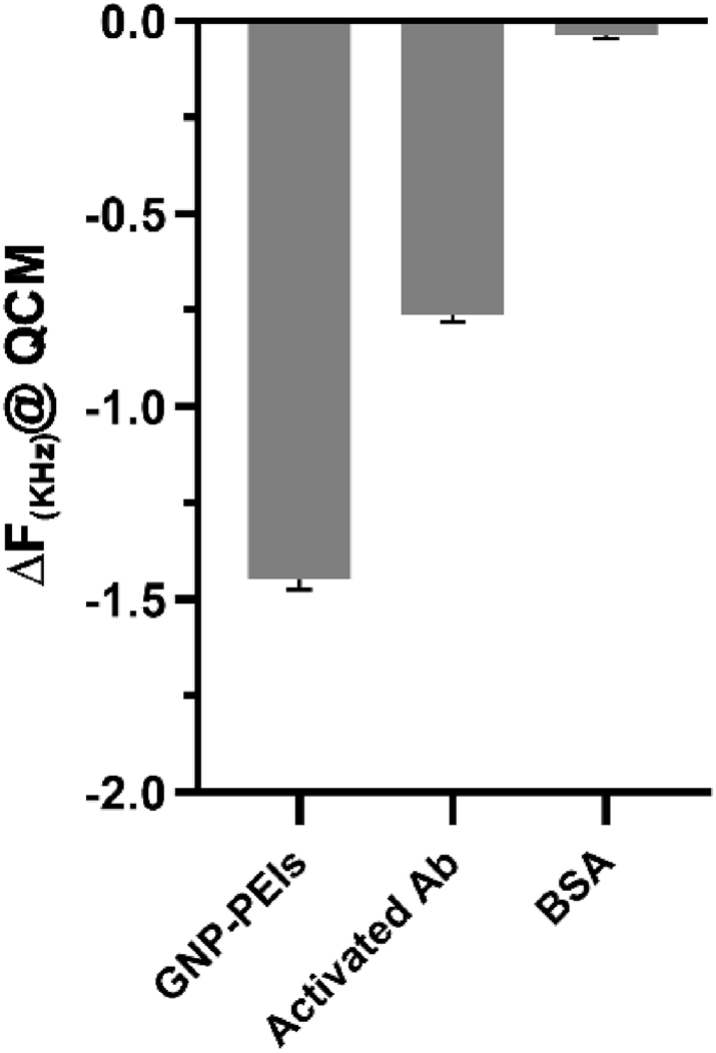
The observed frequency shifts based on individual mass deposition on the QCM-based immunosensor.

The developed QCM biosensor was able to track frequency shifts, which were proportional to the deposition of various masses on the surface. It is well understood that the frequency shift of the biosensor is always proportional to the amount of the loaded layers on it^47^. Figure 6 shows the frequency changes at each step of surface engineering. It can be seen that with an increase in the deposition mass due to the loading of AuNP-PEIs, activated antibodies, and BSA (as a surface blocker), the frequency decreases as explained by Sauerbrey’s equation. The equation is widely used in QCM and expresses the relationship between resonance frequency changes and mass load changes. It must be mentioned that this equation is applicable when the added mass is smaller than that of quartz crystal and uniformly and rigidly adsorbed on the surface^48^.

### Biosensing assay

#### Analytical measurements

**Figure 7:**
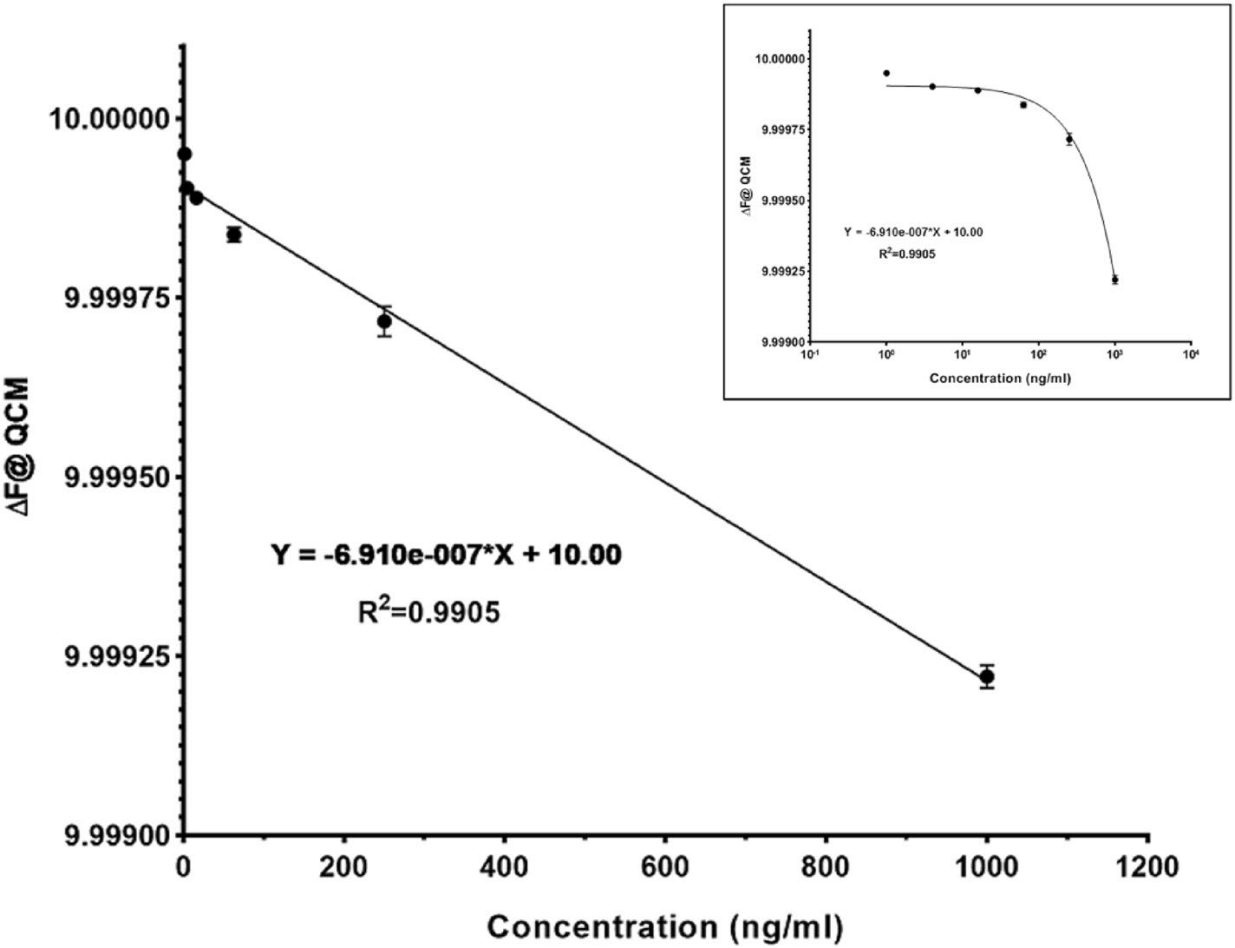
The calibration curve of QCM-based immunosensor based on HBsAg detection. The inset shows the logarithmic scale of data. Data are represented as mean ± SD from three independent experiments.

To determine the LOD and LOQ values, different concentrations of the antigen were added to the QCM-based immunosensor. The frequency shifts were then calculated using Eq. (4) and plotted against concentrations ranging from 1 to 1000 ng/mL (Fig. 7). Statistical analysis revealed a linear regression with a coefficient of determination (R^2^) of 0.9905. The QCM-based immunosensor detected HBsAg with a LOD of 1.49 ng/mL and a LOQ of 4.52 ng/mL, while these values were 0.57 ng/mL and 1.74 ng/mL for the ELISA assay. These values were improved by approximately two-fold compared to our previous works due to the use of different immobilization methods and signal amplification^6,10^. It has been reported that nanomaterials remarkably improve the sensitivity of biosensors when used for surface modification^49^. Taken together, these results demonstrate that the AuNP-PEIs platform has superior and more reliable performance for the detection of antigen–antibody interaction and is hence preferable over the PEI platform for such biosensing in clinical applications^10^. Future studies are warranted to further investigate the effect of potential factors on AuNP-PEIs synthesizing (the operating parameters such as temperature, pH, and concentrations of the reducer) at the antibody immobilization step. Moreover, other functionalized groups on AuNP-PEIs (e.g., thiolated form) can be tested to achieve higher immobilization efficiency and longer stability for targeted applications. The obtained LOD and LOQ values for the present study are comparable to those of developed biosensors for HBsAg detection. For example, Wang et al*.* in 2010 used gold nanorods as an optical transducer in a SPR-based biosensor to screen for HBsAg in buffer, blood serum, and plasma. Their developed biosensor had a LOD of 0.4 IU/mL in a linear range of 0.1–1 IU/mL^50^. In another study, Ehsani et al*.* reported two chemiluminescent immunoassay systems based on CuO nanoparticles and CuO/MWCNT nanocomposites for HBsAg detection with LODs of 1.8 (in a liner range of 3.5 nM to 2.5 μM) and 0.85 ng/mL (in a liner range of 2.2 nM to 5.0 μM), respectively^43^. However, these groups did not report or determined the LOQ values for their biosensors. In 2021, Mohsin et al*.* introduced an electrochemical aptasensor for HBsAg detection. They used a thiol-terminated aptamer with methylene blue as an indicator that covalently linked to a nanoplatform containing gold nanoparticles functionalized reduced graphene oxide. They obtained a LOD of 0.0014 fg/mL and a LOQ of 0.004 fg/mL for HBsAg in a linear range of 0.125–2.0 fg/mL^51^. Although the latter study reported better LOD and LOQ values, nonetheless our nanoplatform is more simple and affordable.

### Correlation and ROC curve analysis

Assessment of diagnostic tests is a significant concern in modern medicine, as it enables the confirmation of the presence or absence of disease and classifies individuals accordingly. In practice, physicians often rely on test results to make the diagnosis. Usually, two important measures for interpreting these results are the PPV and NPV of the test. The PPV and NPV are influenced by various factors, including the test's sensitivity and specificity, as well as the disease's prevalence in the population being tested^52^. It must be noted that two crucial components that determine the validity of a test are sensitivity and 1-specificity relative to the gold standard. When dealing with dichotomous outcomes (positive/negative), the ROC curve and AUC are commonly used as effective measures of accuracy. The ROC curve evaluates the effectiveness of diagnostic tests by calculating sensitivity and specificity across all threshold values, independent of disease prevalence. As a result, this method is highly effective in evaluating the performance of a diagnostic test^53^.

**Figure 8:**
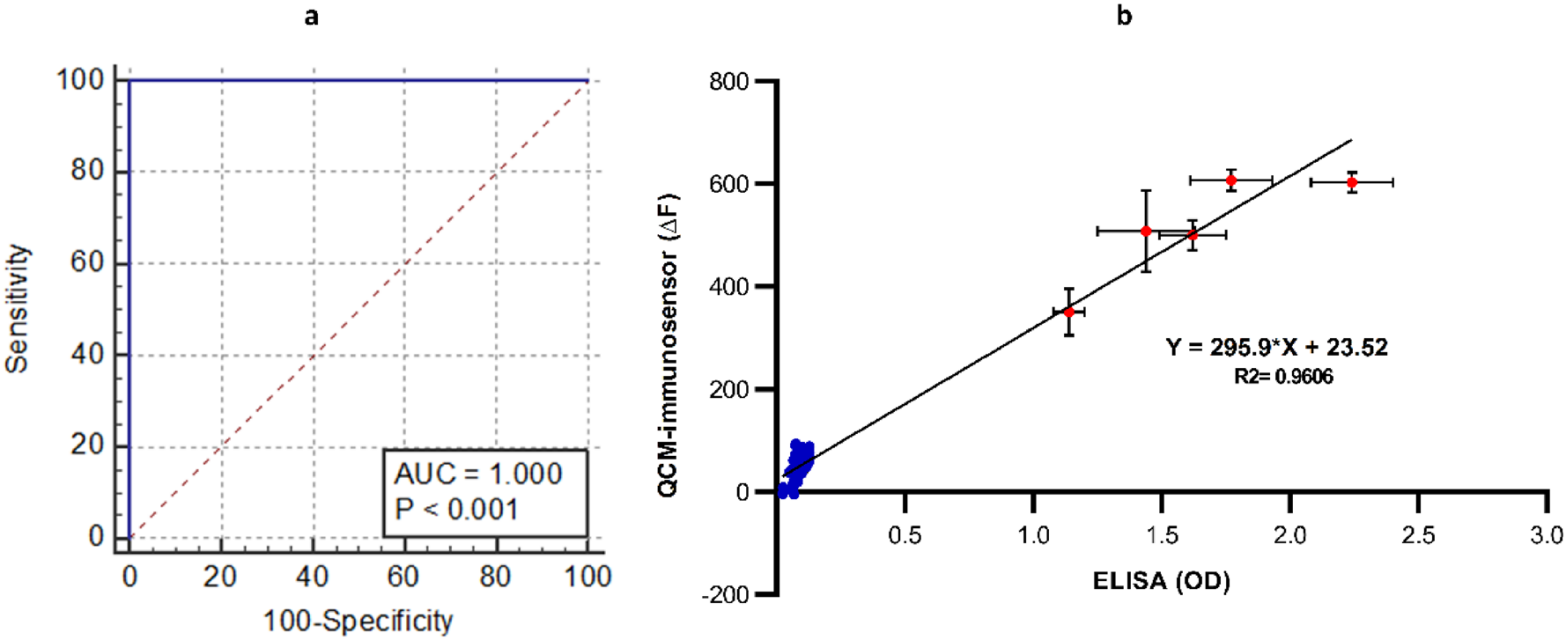
(**a**) ROC analysis of QCM-based immunosensor and (**b**) linear regression analysis between the QCM immunosensor and the ELISA data. The blue points are negative samples and the red points are positive samples.

The AUC of the test was 1.00 with an ideal Cut-off of 292 HZ (Fig. 8). The sensitivity using Cut-off 292 was 100% (95% CI 47.8–100.0) and the specificity was 100% (95% CI 96.2–100.0) with 100% PPV and NPV. The accuracy of the QMC-based Immunosensor was 100% (95% CI 96.38–100.00). The relationship between the QCM immunosensor and the ELISA data was investigated using linear regression analysis. The statistical analysis revealed that the QCM immunosensor and the ELISA data were well-linearly correlated with a coefficient of determination (R^2^) of 0.9606 and a *p*-value less than 0.0001 (Fig. 8).

## Conclusion

QCM-based immunosensors can detect a broad range of biomarkers in clinical samples. In the current study, we benefitted from the advantages of QCM-based immunosensors and AuNP-PEIs nanoparticles (signal amplification) for hepatitis B detection. Our results demonstrated that the developed immunosensor has the potential to be used as a suitable analytic tool for screening, clinical diagnosis, and point-of-care testing with high specificity and sensitivity. However, further investigations are needed to convert the developed immunosensor into a tiny biochip (lab-on-chip) suitable for diagnosing hepatitis B in clinical samples.

### Experimental section

#### Primary reagents

Bovine serum albumin (BSA), 1-Ethyl (3-dimethyl-aminopropyl)-Carbodiimide Hydrochloride (EDC), N-hydroxy-succinimide (NHS), Sodium dodecyl sulfate (SDS), Gold (III) chloride trihydrate (HAuCl4), and branched polyethyleneimine (BPEI, Cat#3880) were supplied by Sigma-Aldrich (USA). Microcon® Centrifugal Filter YM-100 (100 kDa MWCO) was purchased from Millipore, (USA). All solutions were prepared in water for injection (WFI) with a conductivity of not less than 18.2 MΩ cm^-1^. Phosphate buffered saline solution (PBS, pH 7.2) was made using 137 mM NaCl, 2.7 mM KCL, 8.0 mM Na_2_HPO_4_, and 1.5 mM KH_2_PO_4_. Piranha solution was made using 98% H_2_SO_4_ and 30% H_2_O_2_ at a ratio of 3:1 as a washing reagent. HBsAg with a purity of more than 97% was obtained from the Pasteur Institute of Iran. Purified anti-HBsAg Antibody as a receptor element in the QCM-based immunosensor was purchased from Biolegend, Inc. (San Diego, CA, USA). The dialysis tubing (cellulose membrane molecular weight cut-off of 14 kDa) was acquired from Sigma-Aldrich. Commercial ELISA kit was procured from Diapro Company. The piezoelectric quartz crystals (10 MHz AT-cut, OpenQCM, Novaetech S.r.l. Pompeii, Italy) were placed between 2 gold electrodes for electrical connection. The QCM electrode was flat and carefully polished. The adhesion intrinsic of the gold electrode on the QCM electrode (Φ = 6 mm) was enhanced by using a substrate of titanium. Human serum samples (n = 100) were gifted from the Hepatitis and HIV Laboratory, Pasteur Institute of Iran (Tehran, Iran).

### QCM-based immunosensor preparation and characterization

#### Preparation and characterization of AuNP-PEIs

**Figure 9:**
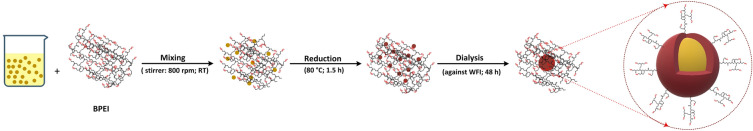
Schematic illustration of the AuNP-PEIs synthesis process.

At first, 1 mL of PEI solution (0.25% w/v in WFI) as a reducing and capping agent was added to 10 mL of HAuCl_4_ solution (2.5 mmol/L) under continuous stirring with a magnetic stirrer (800 rpm) at room temperature (RT). Then, the yellowish prepared mixture was heated up from RT to 80 °C and then kept at 80 °C for 1.5 h^20^. Finally, the resulting purple mixture was cooled to RT. The color conversion step from yellow to purple signified the AuNP-PEIs formation. The final purple product was purified by the dialysis membrane against WFI (three times for 48 h) to eliminate the unreacted chemicals. Finally, the prepared AuNP-PEIs were stored at 4 ^◦^C and sonicated before use (Fig. 9). Physicochemical properties of AuNP-PEIs were characterized via ICP-MS (VISTA-PRO), UV–visible spectroscopy (BioTek, Power wave XS. USA), DLS (Malvern zeta sizer Nano ZS), FE-SEM (TESCAN MIRA III), and ATR-FTIR spectroscopy (Thermo. AVATAR. USA).

#### Coating and characterization of AuNP-PEIs on the gold electrode surface

**Figure 10:**
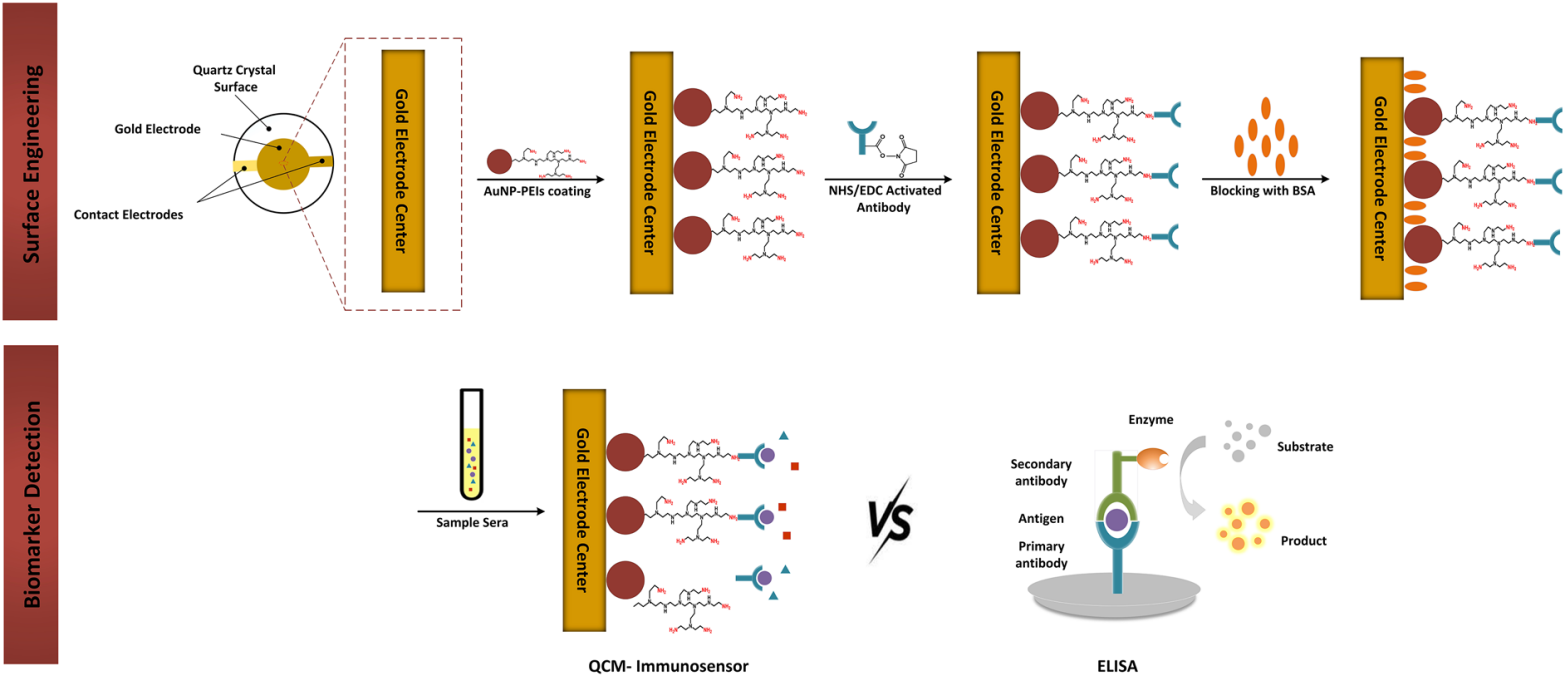
Schematic illustration of the surface engineering and detection process.

Piranha solution was used for 5 min to clean the gold electrode chip. Then, the cleaned surface was washed with WFI and ethanol followed by drying under a nitrogen stream. This procedure removed the contaminants on the chip for better performance of the subsequent layering. In the following, 10 µL of AuNP-PEIs were first seeped onto the cleaned chip and incubated at RT for 24 h. The unbound AuNP-PEIs were then rinsed twice with PBS and WFI. After drying in the ambient, the modified chip was used for antibody immobilization (Fig. 10). The modified surface was characterized chemically by FE-SEM, Energy-dispersive X-ray spectroscopy (EDS) (TESCAN MIRA III), EDS Mapping, and atomic force microscopy (AFM) (ARA-AFM, Ara-Research, Iran). As a static requirement, the surface stability was studied by monitoring the frequency shifts on the gold electrode for 30 days at RT, and WCA was evaluated by measuring the surface hydrophilicity using an optical camera (Dino-Lite, China).

#### Anti-HBsAg antibody immobilization

As described in our previous study, the covalent binding of antibodies on the surface finally leads to better performance of the QCM biosensor ^40^. Therefore, we used EDC/NHS activation protocol to activate the carboxyl groups of antibodies in the form of NHS ester which later could bind to the amine groups of PEI via amide bonds. Moreover, response surface methodology (RSM) was used to optimize the immobilization step. By using Design Expert 11 software, the effects of reaction and immobilization times on the antibody immobilization yield were modeled by a faced center-central composite design (α = 1). Three replicates of the center, axial, and factorial points were used to estimate the pure error of the design. The resulting runs (27 experiments) were performed randomly and the immobilization yield of each experiment was calculated according to Eq. (2). To activate the terminal carboxylic groups on the antibody, the antibody solution (50 pM) was mixed with EDC/NHS solution (25 mM EDC and 50 mM NHS that were prepared in 50 mM MES buffer, adjusted to pH 6) at a ratio of 1:5, gently stirred, and incubated at 4 °C for 20, 25, and 30 min (as the activation time, denoted as AT). The activated antibodies were transferred to the micron tubes and centrifuged at 12,000 g for 5 min to ensure the removal of any residual unbound reagents or buffer components. The buffer exchange step with phosphate buffer was repeated four times. Finally, the activated antibodies (with a final concentration of 40 pM in 1 M phosphate buffer, pH 7.2) were stored at 4 °C and used for the immobilization step. In the next step, 10 µL triggered antibodies were added to the functionalized QCM gold electrode for 90, 105, and 120 min (as immobilization time, denoted as IT). The yield of antibody immobilization (target response) was determined by measuring the amount of the anti-HBsAg antibodies in the solutions before (feed solution) and after (unbound) the immobilization process by NanoDrop at the wavelength of 280 nm (BioTek, Power wave XS (USA)) according to Eq. (2) ^54^.

2 $${\text{Immobilization Yield }}\left( {\text{\%}} \right) = \frac{{{\text{OD}}_{{\text{feed solution of antibody}}} - {\text{ OD}}_{{\text{unbound antibody}}} }}{{{\text{OD}}_{{\text{feed solution of antibody}}} }}$$

In the following, BSA (0.5 mg/mL, in PBS, 1 h, 4 °C) was used as a blocker on the prepared chips to cover the unoccupied surface (Fig. 10). At the end of each step, the unbound or excess materials were first removed by PBS and WFI rinsing, and then, the resonant frequencies were determined with an oscillator and monitored with a personal computer ^10,55^. Hence, Sauerbrey’s equation (Eq. 3) calculates this relationship between the loaded mass and the resonance frequency for each step^56,57^.

3 $$ \Delta F = - 2.3 \times 10^{ - 6} { }\frac{{f_{0}^{2} \Delta M}}{{{\text{A}}\sqrt {Pq\mu q} }} $$

where ∆F (Hz) is the observed frequency shift based on mass deposition on the crystal (Hz), F0 is the basic frequency of piezoelectric quartz, A is the piezoelectric activity geometrical area (cm^2^), and ∆M is the mass changes (g). While Pq and µq terms are the quartz density (g × cm^-3^) and the shear modulus of quartz (g × cm^-1^ × s^-2^), respectively.

#### Analytical measurements of QCM-based immunosensor

For detecting the antibody-antigen interaction, serially diluted solutions (1–1000) were prepared from HBsAg standard solution (1 µg/mL, in PBS). Then, 10 μL of the standards was added to the QCM-based immunosensor and incubated for 45 min. The electrodes were then washed three times with WFI and PBS to remove unreacted antigens. Finally, the shift frequency of interaction was measured by a homemade oscillator. After transferring data to the microcontroller Arduino Nano, the result was displayed on a PC. The resonant frequency was registered before (F_1_) and after (F_2_) loading of 10 µL sample on the QCM-based immunosensor. Frequency differences were calculated using Eq. (4).

4 $$ \Delta F_{{\left( {{\text{standard}}/{\text{sample}}} \right)}} = F_{2} - F_{1} $$

In the following, LOD and LOQ values of the QCM**-**based immunosensor were measures to determine the sensitivity of the analytical method. In this context, the LOD and LOQ are expressed using the following Eq. (5) ^10,58,59^:

5 $$ L = F \frac{{\upsigma }}{S} $$

In this equation, L represents the LOD or LOQ values. F is a factor that equals 3.3 for LOD and 10 for LOQ. σ corresponds to the standard deviation of the blank (not containing the analyte) response. S represents the slope of the calibration curve.

### HBsAg measurement in biological samples

#### Preparation of specimens

Human sera samples (n = 100; n _positive_ = 5, n _negative_ = 95) were gifted from the reference laboratory of the Hepatitis and AIDS department of the Pasteur Institute of Iran during 2021–2022.

#### QCM-based immunosensor assay

The positive sera samples were initially diluted with PBS (0.1 M, pH 7.4) at a 20-fold dilution. The purpose of this dilution was to bring the samples within the dynamic range of measurements. The diluted samples were analyzed by ELISA kit (gold standard assay) and QCM-based immunosensor as follows (Fig. 10). Finally, the shift frequency of interaction was measured by a homemade oscillator. After transferring data to the microcontroller Arduino Nano, the result was displayed on a PC. The frequency changes for the sera samples were then calculated using Eq. (4).

#### ELISA assay

The ELISA was performed according to the manufacturer’s protocol. In brief, 150 µL of each sample (including a positive control, a negative control, and human sera) was added to a 96-well micro plate, mixed with 100 µL of the diluted enzyme conjugate, and incubated for 120 min at 37 ℃. After washing, 200 µL of the TMB substrate (contains 50 mM citrate–phosphate buffered solution at pH 3.5–3.8, 4% dimethylsulphoxide, 0.03% tetra-methyl-benzidine (TMB) and 0.02% hydrogen peroxide (H_2_O_2_)) was added and the mixture was incubated for 30 min at RT in the dark condition. The reaction was stopped by adding 100 µL of sulfuric acid. Finally, the optical density (OD) was measured at a wavelength of 450 nm/620 nm using an ELISA reader (BIOHIT; Sartorius Biohit Liquid Handling Oy, Kajaani, Finland). For consistency with previous studies and to facilitate comparison of results, we used a Sample/Cut-Off value of 1.1 as the Cut-off for the ELISA, which was recommended by the kit manufacturer. This means that a Sample/Cut-Off value ≥ 1.1 was considered positive for HBsAg detection.

### Statistical analysis

The normal distribution of ELISA and QCM data was tested using Shapiro–Wilk test. A one-sample T-test was used to compare the optimum predicted and empirical values. The coefficient of determination (R^2^) were calculated through linear regression analysis. ROC analysis was used to determine the Cut-Off and calculate the sensitivity, specificity, PPV, and NPV of the QCM-based immunosensor. Statistical analysis was done using SPSS (Version 21) and Med Calc Version 20.216 software. For all statistical analyses, the level of significance was set to a *p*-value less than 0.05 with a confidence interval of 95%. Other graphs were plotted using GraphPad Prism 5.0. The impact of the main factors affecting the immobilization yield was modeled and optimized using Design Expert 11.

### Supplementary Information

Supplementary Tables.

## Data Availability

All data generated or analyzed during this study are included in the published article, and or are available from the corresponding author on reasonable request.
