# Supplementary material for: Signal amplification of a quartz crystal microbalance immunosensor by gold nanoparticles-polyethyleneimine for hepatitis B biomarker detection
Source: Sci Rep. 2023 Dec 9;13:21851. doi: 10.1038/s41598-023-48766-2 (PMC10710426; doi:10.1038/s41598-023-48766-2)
Supplement: Supplementary file 1 — Supplementary Tables. [file 41598_2023_48766_MOESM1_ESM.pdf]

Supplementary information

Table S_1. The designed experimental matrix and the corresponding results for target response.

Run	Factor (A): Antibody Activation Time (min)	Factor (B): Antibody Immobilization Time (min)	Response (Y): Immobilization Yield (%)
	20	90	73.8
**2**	20	90	76.12
**3**	20	90	74.5
**4**	40	90	68.5
**5**	40	90	66.42
**6**	40	90	72.1
**7**	20	120	82.2
**8**	20	120	81.6
**9**	20	120	82.5
**10**	40	120	72.16
**11**	40	120	71.67
**12**	40	120	68.92
**13**	20	105	78.13
**14**	20	105	77.25
**15**	20	105	80.15
**16**	40	105	71.5
**17**	40	105	52.3*
**18**	40	105	70.1
**19**	30	90	88.53
**20**	30	90	86.12
**21**	30	90	87.6
**22**	30	120	94.5
**23**	30	120	94.41
**24**	30	120	93.19
**25**	30	105	91.36
**26**	30	105	88.74
**27**	30	105	89.32

*Outlier

Table S2. The ANOVA results for the quadratic model.

Source	Sum of Squares	df	Mean Square	F-value	p-value	
**Model**	1917.62	4	479.40	219.38	< 0.0001	significant
**A-Activation Time**	291.84	1	291.84	133.55	< 0.0001	
**B-Immobilization Time**	125.14	1	125.14	57.26	< 0.0001	
**AB**	21.74	1	21.74	9.95	0.0048	
**A^2**	1522.98	1	1522.98	696.93	< 0.0001	
**Residual**	45.89	21	2.19			
**Lack of Fit**	6.81	4	1.70	0.7403	0.5775	not significant
**Pure Error**	39.08	17	2.30			
**Cor Total**	1963.51	25				

Table S3. One-sample T-test results for comparing experimental immobilization yields against the predicted optimum values.

	Experimental. Value (mean± SD)	Test Value (predicted optimum yield)	t	df	Sig. (2-tailed)	Mean Difference	95% Confidence Interval of the Difference	
							Lower	Upper
**Immobilization yield**	94.03 ± 0.073	93.5	1.262	2	0.334	0.533	-1.284	2.351
